# Association between self-reported evidence-based medicine competencies and prescribing of drugs without scientific evidence against mild COVID-19 among recently graduated physicians in Peru

**DOI:** 10.1016/j.heliyon.2023.e15366

**Published:** 2023-04-08

**Authors:** Daniel Fernandez-Guzman, Brenda Caira-Chuquineyra, Fiorella Baca-Rondan, Maria Cristina Yucra-Sosa, Fabricio Ccami-Bernal, David R. Soriano-Moreno, Wendy Nieto-Gutierrez, Vicente A. Benites-Zapata

**Affiliations:** a Universidad Cientifica del Sur, Lima, Peru; b Facultad de Medicina, Universidad Nacional de San Agustín de Arequipa, Arequipa, Peru; c Escuela Profesional de Medicina Humana, Universidad Nacional de San Antonio Abad del Cusco, Cusco, Peru; d Unidad de Investigación Clínica y Epidemiológica, Escuela de Medicina, Universidad Peruana Unión, Lima, Peru; e Unidad para la Generación y Síntesis de Evidencias en Salud, Universidad San Ignacio de Loyola, Lima, Peru

**Keywords:** COVID-19, Ivermectin, Hydroxychloroquine, Azithromycin, Drug prescriptions: physicians, Evidence-based medicine, Peru (Source: MeSH)

## Abstract

**Objective:**

To evaluate the association between self-rated evidence-based medicine (EBM) competencies and the prescription of drugs without scientific evidence against mild COVID-19 (present with any of the signs and symptoms of COVID-19 but who do not have shortness of breath, dyspnea, or abnormal chest imaging) among recently graduated physicians in Peru.

**Methods:**

We conducted an analytical cross-sectional study where we evaluated a non-probability sample of recently graduated physicians during June and July 2021 (end of second wave of COVID-19 in Peru). Self-rated EBM competencies were assessed by four domains (formulation of a clinical question, search, analysis, and application) using a Likert scale with scores from zero to four (“Very inadequate” = 0, to “Very Adequate” = 4), it was considered as “Adequate” if the score was three or four. In addition, the variable “General competence on EBM” was rated as “Adequate” if in all domains evaluated it presented an adequate self-rating. For the outcome, drug prescription, we considered the use of ivermectin, azithromycin, other antibiotics, hydroxychloroquine, dexamethasone, and anticoagulants (drugs with no efficacy demonstrated for patients with mild COVID-19). To assess the association, we used Poisson regression models with robust variances and obtaining crude (cPR) and adjusted (aPR) prevalence ratios with their 95% confidence intervals (95%CI).

**Results:**

Of a total of 239 physicians included 70.7% prescribed at least one drug without scientific evidence. A total of 51.1% reported adequate ratings in all evaluated domains of EBM. Self-rating the “Clinical Question Formulation” competency as adequate was associated with a lower frequency of prescribing medications for mild COVID-19 (aPR: 0.93; 95% CI: 0.91–0.95). While self-rating as adequate the competency of “Identify possible implications of investigations” was associated with an increase in the prescription of such drugs (aPR: 1.14; 95% CI: 1.09–1.20). Additionally, self-rating all domains as adequate were associated with less prescription (aPR: 0.93; 95% CI: 0.90–0.96).

**Conclusion:**

Seven out of ten recently graduated physicians prescribed some type of medication without scientific evidence to treat patients with mild COVID-19. Having adequate self-perceived EBM competencies was associated with a lower frequency of prescribing medications without scientific evidence to manage patients with mild COVID-19.

## 1 Introduction

The COVID-19 pandemic has generated a public health crisis, causing high morbidity and mortality, especially in developing countries [1]. This was the case in Peru, where the health system was overwhelmed by the high demand for patients with COVID-19 [2,3], which, together with the increase in unreliable information on the management of the disease, led to a greater impact on the health crisis [4].

The publication of studies with small samples, without a control group, and with major methodological flaws, led many health systems and, therefore, physicians to use drugs to reduce the transmissibility and mortality of COVID-19 in all stages of severity [5,6]. Even though world guidelines rejected and recommended the non-use of some drugs such as ivermectin, azithromycin, and hydroxychloroquine, among others [[7], [8], [9]], many countries, including Peru, continued to prescribe their use for management, even in asymptomatic patients [[10], [11], [12]]. This led to adverse effects and drug-drug interactions, which increased the risk of developing a worse outcome of the disease [13,14].

On the other hand, some previous studies have reported that competencies in Evidence-Based Medicine (EBM) play an important role in the correct decision-making in disease management [15,16], for example, it has been shown that the acquisition of EBM competencies can improve the ability and motivation of health professionals to use research evidence to review and improve clinical practice [17]. Therefore, a poor application of evidence for a drug prescription in COVID-19 could be due to a lack of training in EBM-related competencies. Especially because in Peru, there is a history of few medical schools teaching courses related to EBM. In addition, the programs of these courses do not cover all the topics necessary to ensure that students acquire the essential competencies related to EBM [18]. In addition, many Peruvian physicians present conflicts between evidence-based practice and prioritizing experiential learning [19], including, in years before the pandemic, a poor perception of EBM competencies in Peruvian physicians has been identified [20].

For this reason, it is relevant to evaluate EBM, especially in more vulnerable populations, such as recent graduates, whose experience in the clinical field is less. The present study aimed to evaluate the association between self-rated EBM competencies and the prescription of drugs without scientific evidence against mild COVID-19 among recently graduated Peruvian physicians. The results of this study could provide important insights into the role of EBM in decision-making and inform the development of targeted training and education programs to improve EBM competencies among physicians. Ultimately, this could lead to better outcomes for patients and help to mitigate the impact of COVID-19 on the health system in Peru.

## 2 Methods

### 2.1 Study design and population

We conducted an analytical cross-sectional study, where the study population was recently graduated Peruvian physicians. The inclusion criteria were to be a Peruvian physician performing the rural and marginal urban health service (SERUMS) during the first and second periods of 2020 (SERUMS 2020-I and 2020-II). Those physicians who did not give informed consent and/or incorrectly filled out the questions related to the outcome were excluded.

#### 2.1.1 Context

SERUMS is a social and health service program aimed at the community, which is carried out by health science professionals, primarily in low-income and remote areas of Peru, following the provisions of Law No. 23330 [21]. This service is oriented to provide health care under a preventive-promotional public health approach, for 12 months in health facilities of the first levels of care. Two calls for SERUMS are made annually [22]. For the year 2020, the duration of the first call (SERUMS-I) was reduced to only 10 months due to the health emergency that was experienced as a result of the COVID-19 pandemic, beginning in July 2020 and ending in April 2021 [23]. The second edition (SERUMS-II) was 12 months, beginning in October 2020 and ending in September 2021 [24]. This study was conducted between 20 June and 20 July 2021, a period in which the second wave of COVID-19 was in recession in Peru [25].

### 2.2 Sample and sampling

During the period evaluated, 3,632 physicians were registered in SERUMS-I and II [26,27]. Due to the lack of a means of mass communication with all SERUMS physicians, we enrolled them by means of a non-probabilistic snowball sampling. We calculated a reference sample size, considering a difference in the proportion of prescribing drugs without scientific evidence of 15% (70% vs. 55%), a 95% confidence level, and a power of 80%. Thus, the calculated sample size was 326 physicians. Since we did not have any background on EBM and prescription competence, we considered the study by Soriano-Moreno DR et al. [28], to estimate the percentage of patients with mild COVID-19 who consumed some type of medication in Peru during 2020 (first wave of cases) and had received medical information (∼70%, assuming that the physicians had inadequate competencies in EBM), and we considered a proportion of 55% (proportion of patients who consumed medication and who had received medical information by physicians with adequate competencies in EBM) trying to be conservative with the sample size calculation.

A small acceptance rate was expected since it was a virtual survey, applied when the physicians were completing their SERUMS. Because of this, only 73.3% of the target population (n = 239) was surveyed. Given this scenario, we decided to evaluate the statistical power for the self-rated EBM competencies, finding that the power for the associations between the dependent variable (prescribing medications without scientific evidence) and the self-rated competencies, “Finding quality scientific research,” “Understanding scientific research information,” “Judging the quality of scientific research information,” and “Applying evidence to practice” was 6.4%, 12.3%, 3.3%, 3.3%, and 5.1%, respectively. Likewise, the statistical power for the overall competency on EBM was 31.9%.

### 2.3 Procedure

The authors and collaborators sent a Google questionnaire to physicians, via social networks (WhatsApp, and Facebook, among others), emails, and Facebook and Telegram groups for a period of 31 days (from June 20th to July 20th, 2021, end of second wave of COVID-19 in Peru).

### 2.4 Questionnaire

A questionnaire was structured using the Google forms platform, which was developed from previous studies that evaluated EBM competencies [[29], [30], [31], [32], [33], [34]].

The questionnaire consisted of three sections: 1) general data 2) prescription patterns for treating COVID-19, and 3) self-perceived EBM competencies. Additionally, questions were asked about the source of information used to learn about COVID-19 and the perceived barriers limiting the application of EBM in their clinical practice.

The proposed questions were structured based on previous studies [35,36] and validated by two medical epidemiologists. In addition, we evaluated that the questions were adequately understood with a pilot sample of 10 recently graduated physicians. Furthermore, Cronbach's Alpha was calculated, post-application of the questionnaire resulting in a good validity of the overall survey (0.79), as well as for the second section on prescribing (0.87) and the third section on self-rated EBM competencies (0.77). The complete questionnaire can be found in supplementary material.

### 2.5 Dependent variable

Prescription of medications without scientific evidence for patients with mild COVID-19, was considered as “Never did” in case the physician did not prescribe in any month during their SERUMS the following medications: ivermectin, azithromycin, hydroxychloroquine, dexamethasone, oral anticoagulants, other antibiotics, or chlorine dioxide. We chose those drugs because at the time of the pandemic, they were demonstrably ineffective in treating mild COVID-19 [37]. On the other hand, it was considered as “Yes he did” when the physician reported having prescribed any of the aforementioned medications during the months that his SERUMS lasted.

To define a patient with mild COVID-19, we provided a brief description to respondents as part of the question. We used the definition recommended by the National Institutes of Health (United States), which defines mild COVID-19 as those who present with any of the signs and symptoms of COVID-19 (e.g., fever, cough, sore throat, malaise, headache, muscle pain, nausea, vomiting, diarrhea, loss of taste and smell) but who do not have shortness of breath, dyspnea, or abnormal chest imaging [38].

### 2.6 Independent variable

A previous consensus grouped EBM competencies into six domains: a) introduction, b) formulation of a clinical question, c) search, d) analysis, e) application, and f) evaluation [39]. For the present study, four domains were evaluated (neither the “introduction” nor the “evaluation” domain were included because the introduction contained general concepts that could not be evaluated, while for the “evaluation” domain there was no follow-up of the clinical outcomes of the patients attended) through the following question: “How would you rate your competencies on the following EBM topics? a) Defining and asking a scientific question (formulation of a clinical question domain); b) Finding quality scientific research (search domain); c) Understanding scientific research information (analysis domain), d) Judging the quality of scientific research information (analysis domain), and e) Identifying possible implications of scientific research (analysis domain); and f) Applying evidence to practice (application domain)”.

The competencies on EBM were self-rated by the respondents according to a Likert scale that had as options “Very inadequate” = 0, “Inadequate” = 1, “Neutral” = 2, “Adequate” = 3, “Very Adequate” = 4. For the present study, it was recategorized as “Inadequate” if the competencies were rated with a lower score equal to two, while it was considered as “Adequate” if the score was three or four. In addition, the variable “General competence on EBM” was constructed which it was rated as “Adequate” if in all domains evaluated it presented an adequate self-rating.

### 2.7 Other variables

The following covariates were included: sociodemographic characteristics such as age tertiles (22–25 years, 26–27 years, and 28–38 years), sex (female, male), marital status (single y married/cohabiting), type of university where studied (public and private). Also considered were month of SERUMS start (July 2020 and October 2020), region where SERUMS is performed (Coast, Highlands, and Jungle), zone where SERUMS was performed (rural, and non-rural), category of health center where SERUMS was performed (I-2, and I-3 o I-4), COVID-19 personal history (no, and yes), received EBM training (no, and yes), belief that there is sufficient evidence for prescribing medication for mild COVID-19 (no, and yes), and belief that at least one drug is effective against mild COVID-19 (no, and yes).

### 2.8 Statistical analysis

Data analysis was performed using Stata v16.0. A descriptive analysis was performed using absolute and relative frequencies for the categorical variables and mean with standard deviation for the numerical variable (age). To evaluate differences between groups, we used the Chi-square test and Student's t-test.

To avoid overestimation of the effect of the association between the variables of interest [40], we performed Poisson family generalized linear models (GLM) with logarithmic link function to evaluate the association of interest, as well as the association with each of the components of the EBM competencies. In this way we report the crude (cRP) and adjusted (aRP) Prevalence Ratios with their respective 95% confidence intervals (95%CI). We have considered as a cluster the region where SERUMS was performed (coast, highlands and jungle) for the Poisson regression. For the adjusted model, we used an epidemiological approach [41], including the following confounding variables: age, sex, current marital status, type of university of undergraduate study, period of SERUMS initiation, area of residence, category of the health center where he/she works and personal history of COVID-19. For all statistical tests, values of p < 0.05 were considered statistically significant.

Finally, we evaluated multicollinearity in the fitted regression model using the variance inflation factor (VIF), where a value > 10 determined multicollinearity among the variables; however, all values obtained were less than 10.

## 3 Ethical issues

The study protocol was approved by the ethics committee of the Universidad Peruana Unión (code: 2021-CEUPeU-0058) and was also registered in the Health Research Projects Platform (PRISA) of Peru (code: EI00000001774). All participants gave informed consent and the survey was anonymous and confidential.

## 4 Results

A total of 239 recently graduated physicians were evaluated, of whom 194 (81.2%) performed SERUMS-I and 45 (18.8%) SERUMS-II. Among the participants, the average age was 27.0 ± 2.6 years, 55.7% were women and 53.1% came from private universities. Among the characteristics related to SERUMS, 72% performed in the highland's region, 74.5% in a rural area, and 69.5% in a category I-2 health center. On the other hand, 87.5% reported having received training in EBM, 25.9% believe that there is sufficient evidence to prescribe drugs for mild cases of COVID-19 and 69.9% of the surveyed physicians believe that at least one drug is effective against mild COVID-19 (Table 1).

**Table 1:** Characteristics of physicians evaluated according to history of prescribing drugs without scientific evidence against COVID-19.

Characteristics	N (%)	Prescription of drugs without scientific evidence		
		Never prescribed	Ever prescribed	p-value*
	239 (100%)	70 (29.3%)	169 (70.7%)	
Age	27.0 ± 2.6	27.0 ± 2.3	27.0 ± 2.7	0.987
22–25 years	78 (32.6%)	22 (28.2%)	56 (71.8%)	0.868
26–27 years	85 (35.6%)	24 (28.2%)	61 (71.8%)	
28–38 years	76 (31.8%)	24 (31.6%)	52 (68.4%)	
Sex				
Female	133 (55.7%)	35 (26.3%)	98 (73.7%)	0.258
Male	106 (44.3%)	35 (33.0%)	71 (67.0%)	
Marital Status				
Single	221 (92.5%)	69 (31.2%)	152 (68.8%)	**0.021**
Married or cohabiting	18 (7.5%)	1 (5.6%)	17 (94.4%)	
Type of university where studied				
Public	112 (46.9%)	37 (33.0%)	75 (67.0%)	0.232
Private	127 (53.1%)	33 (26.0%)	94 (74.0%)	
Month SERUMS started				
July 2020	194 (81.2%)	57 (29.4%)	137 (70.6%)	0.948
October 2020	45 (18.8%)	13 (28.9%)	32 (71.1%)	
Region where SERUMS is performed				
Coast	47 (19.7%)	14 (29.8%)	33 (70.2%)	0.634
Highlands	172 (72.0%)	52 (30.2%)	120 (69.8%)	
Jungle	20 (8.4%)	4 (20.0%)	16 (80.0%)	
Zone where SERUMS was performed				
Rural	178 (74.5%)	50 (28.1%)	128 (71.9%)	0.487
Non-Rural	61 (25.5%)	20 (32.8%)	41 (67.2%)	
Category of health center where SERUMS was performed				
I-2	166 (69.5%)	49 (29.5%)	117 (70.5%)	0.906
I-3 o I-4	73 (30.5%)	21 (28.8%)	52 (71.2%)	
COVID-19 Personal History				
No	160 (67.0)	57 (35.6%)	103 (64.4%)	**0.002**
Yes	79 (33.1)	13 (16.5%)	66 (83.5%)	
Received EBM training				
No	30 (12.6%)	9 (30.0%)	21 (70.0%)	0.927
Yes	209 (87.5%)	61 (29.2%)	169 (70.7%)	
Belief that there is sufficient evidence for prescribing medication for mild COVID-19				
No	177 (74.1%)	70 (39.6%)	107 (60.5%)	< **0.001**
Yes	62 (25.9%)	0 (0.0%)	62 (100.0%)	
Belief that at least one drug is effective against mild COVID-19				
No	72 (30.1%)	38 (52.9%)	34 (47.2%)	< **0.001**
Yes	167 (69,9%)	32 (19,2%)	135 (80,8%)	

* Calculated by Chi-square test of independence for crossover of categorical variables and Student's t-test for the numerical variable age. p values < 0.05 are in bold.

Regarding sources of information on COVID-19, 87.1% reported using Scielo, 82.4% used PubMed and 77.9% used the recommendations of the Peruvian Ministry of Health (MINSA). Of the information sources requiring a subscription, approximately 59.0% did not use Scopus and 64.5% did not use Web of Science (Fig. S1).

Within the EBM competencies, most of the physicians surveyed rated as adequate their ability to define and ask a scientific question (75.3%), find quality scientific research (80.3%), understand scientific research information (83.7%), judge the quality of scientific research information (69.5%) and to apply scientific evidence to medical practice (87.9%). However, when assessing overall competence, only 51.1% presented adequate ratings in all EBM domains (Table 2).

**Table 2:** Self-rated competencies on EBM in physicians evaluated according to prescribing history without scientific evidence against COVID-19.

Characteristics	N (%)	Prescription of drugs without scientific evidence		
		Never prescribed	Ever prescribed	p-value*
	239 (100%)	70 (29.3%)	169 (70.7%)	
Define and conduct a scientific question				
Not adequate	59 (24.7%)	14 (23.7%)	45 (76.3%)	0.280
Adequate	180 (75.3%)	56 (31.1%)	124 (68.9%)	
Finding quality scientific research				
Not adequate	47 (19.7%)	15 (31.9%)	32 (68.1%)	0.659
Adequate	192 (80.3%)	55 (29.3%)	137 (70.7%)	
Understanding scientific research information				
Not adequate	39 (16.3%)	13 (33.3%)	26 (66.7%)	0.544
Adequate	200 (83.7%)	57 (28.5%)	143 (71.5%)	
Judging the quality of scientific research information				
Not adequate	73 (30.5%)	21 (28.8%)	52 (71.2%)	0.906
Adequate	166 (69.5%)	49 (29.5%)	117 (70.5%)	
Identifying the possible implications of scientific research				
Not adequate	64 (26.8%)	22 (34.4%)	42 (65.6%)	0.296
Adequate	175 (73.2%)	48 (27.4%)	127 (72.6%)	
Apply evidence to practice				
Not adequate	29 (12.1%)	8 (27.6%)	21 (72.4%)	0.83
Adequate	210 (87.9%)	62 (29.5%)	148 (70.5%)	
General competences in EBM				
Not adequate	117 (49.0%)	29 (24.8%)	88 (75.2%)	0.134
Adequate	122 (51.1%)	41 (33.6%)	81 (66.4%)	

EBM: Evidence-Based Medicine.*Calculated by Chi2 test of independence. *P* values < 0.05 are in bold.

The majority of respondents who self-rated all competencies in EBM (general competence) as adequate were those aged 22–25 years (57.7%; p = 0.185), those who graduated from a public university (55.4%; p = 0.211), those residing in non-rural areas (60.7; p = 0.062) and those who thought that there is not at least one drug effective against mild COVID-19 (59.7%; p = 0.078) (Table 3).

**Table 3:** Characteristics of the physicians evaluated according to their self-rated competence in Evidence-Based Medicine (EBM).

Characteristics	General competences in EBM		
	Not adequate	Adequate	p-value*
	117 (49.0%)	122 (51.1%)	
Age	27.1 ± 2.8	26.9 ± 2.5	0.386
22–25 years	33 (42.3)	45 (57.7)	0.185
26–27 years	48 (56.5)	37 (43.5)	
28–38 years	36 (47.5)	40 (52.5)	
Sex			
Female	68 (51.1)	65 (48.9)	0.451
Male	49 (46.2)	57 (53.8)	
Marital Status			
Single	106 (48.0)	115 (52.0)	0.283
Married or cohabiting	11 (61.1)	7 (38.9)	
Type of university where studied			
Public	50 (44.6)	62 (55.4)	0.211
Private	67 (52.8)	60 (47.2)	
Month SERUMS started			
July 2020	98 (50.5)	96 (49.5)	0.316
October 2020	19 (42.2)	26 (57.8)	
Region where SERUMS is performed			
Coast	23 (48.9)	24 (51.1)	0.58
Highlands	82 (47.7)	90 (52.3)	
Jungle	12 (60.0)	8 (40.0)	
Zone where SERUMS was performed			
Rural	93 (52.3)	85 (47.7)	0.082
Non-Rural	24 (39.3)	37 (60.7)	
Category of health center where SERUMS was performed			
I-2	81 (48.8)	85 (51.2)	0.941
I-3 o I-4	36 (49.3)	37 (50.7)	
COVID-19 Personal History			
No	73 (45.6)	87 (54.4)	0.143
Yes	44 (55.7)	35 (44.3)	
Received EBM training			
No	18 (60.0)	12 (40.0)	0.196
Yes	99 (47.4)	110 (52.6)	
Belief that there is sufficient evidence for prescribing medication for mild COVID-19			
No	85 (48.0)	92 (52.0)	0.626
Yes	32 (51.6)	30 (48.4)	
Belief that at least one drug is effective against mild COVID-19			
No	29 (40.3)	43 (59.7)	0.078
Yes	88 (52.7)	79 (47.3)	

*Calculado por la prueba de independencia Chi-cuadrado para cruce de variables categóricas y t de student para la variable numérica edad. Los valores p < 0,05 están en negrita

**Fig. 1:**
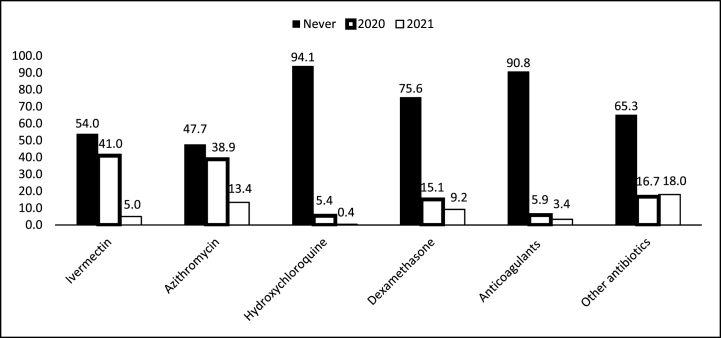
Medications prescribed by the physicians evaluated (n = 239).

Among the drugs prescribed by SERUMS physicians, 5% reported at least once prescribing hydroxychloroquine, 9% prescribed oral anticoagulants, and 24% prescribed dexamethasone. Regarding the most prescribed drugs in 2020, 41% and 39% reported having prescribed ivermectin and azithromycin, respectively. Meanwhile, in 2021, 18% reported prescribing other antibiotics, 13% azithromycin, and 9% dexamethasone (Fig. 1).

The prevalence of prescribing drugs without scientific evidence among SERUMS physicians was 70.7%, with a higher proportion among those who were married or cohabiting (94.4%; p = 0.021) and in those with a personal history of COVID-19 (83.5%; p = 0.002). Likewise, the totality of respondents who have the belief that there is sufficient evidence to prescribe medications for mild COVID-19 and the majority of those who think that at least one medication is effective against mild COVID-19 prescribed medications without scientific evidence (Table 1).

In the adjusted regression model, self-rating as adequate the competency of “Identify possible implications of scientific research” (aPR: 1.14; 95% CI: 1.09–1.20), was associated with a higher frequency of prescribing drugs against mild COVID-19. While self-rating as adequate the competency of “Define and ask a scientific question” (aPR: 0.93; 95% CI: 0.91–0.95) was associated with a lower frequency of prescribing medication to treat cases of mild COVID-19. Concerning overall competence (assessment of all domains together) on EBM, it was found that perceiving it as adequate was associated with a lower frequency of medication prescription (aPR: 0.93; 95% CI: 0.90–0.96) (Table 4).

**Table 4:** Poisson regression models to assess the association between self-rated competencies on EBM and prescribing drugs without scientific evidence against COVID-19 (n = 239).

Characteristics	Crude Model			Adjusted Epidemiological Model *		
	cPR	95% CI	*p-value*	aPR	95% CI	*p-value*
Define and conduct a scientific question						
Not adequate	Ref.			Ref.		
Adequate	0.9	0.87–0.94	< **0.001**	0.93	0.91–0.95	< **0.001**
Finding quality scientific research						
Not adequate	Ref.			Ref.		
Adequate	1.05	0.95–1.15	0.343	1.06	0.96–1.16	0.274
Understanding scientific research information						
Not adequate	Ref.			Ref.		
Adequate	1.07	0.93–1.24	0.344	1.16	0.99–1.35	0.065
Judging the quality of scientific research information						
Not adequate	Ref.			Ref.		
Adequate	0.99	0.92–1.06	0.759	1.02	0.97–1.08	0.383
Identifying the possible implications of scientific research						
Not adequate	Ref.			Ref.		
Adequate	1.11	1.05–1.17	< **0.001**	1.14	1.09–1.20	< **0.001**
Apply evidence to practice						
Not adequate	Ref.			Ref.		
Adequate	0.97	0.84–1.12	0.711	1.03	0.87–1.21	0.724
General competences in EBM						
Not adequate	Ref.			Ref.		
Adequate	0.88	0.82–0.95	**0.001**	0.93	0.90–0.96	< **0.001**

cPR, crude prevalence ratio; aPR, adjusted prevalence ratio.

Prevalence ratios and confidence intervals were calculated by Poisson regression considering the region as a cluster. Values of p < 0.05 are in bold.

*Each component, as well as the overall construct were adjusted for age, sex, current marital status, type of university of undergraduate study, period of SERUMS initiation, area of residence, category of the health center where he/she works and personal history of COVID-19.

Finally, respondents stated that the main reasons for not applying EBM in clinical practice were: lack of access to resources to find quality evidence (43.1%), lack of patient acceptance of new clinical practices (41.8%), lack of logistic resources (41.0%), and lack of education in EBM (38.9%) (Fig. S2).

## 5 Discussion

### 5.1 Main findings

In the present study, we found that seven out of ten newly graduated physicians had prescribed drugs without scientific evidence for mild COVID-19, with azithromycin, ivermectin, other antibiotics, and dexamethasone being the most prescribed during 2020 and 2021. In addition, we evidenced that one out of two physicians self-rated all EBM competencies as adequate, which was associated with a lower frequency of drug prescribing.

### 5.2 Prescription of drugs without scientific evidence

Previous studies have shown that the prescription of non-evidence-based medications for the treatment of COVID-19 is frequent, ranging from 28 to 29% [42,43]. Furthermore, a previous study evaluated the factors influencing medication prescription for COVID-19 [44]. However, to our knowledge, no study has evaluated the relationship between competencies in evidence-based medicine and the prescription of non-evidence-based medications in patients with mild COVID-19. Our results show that seven out of ten newly licensed physicians prescribed some medication without scientific evidence to treat mild cases of COVID-19, a frequency higher than previously reported In our context, such prescription was probably favored because recently graduated physicians can perform certain practices because many people believe it and perform it, mainly their superiors or mentors. This has probably been favored by the low training in EBM competencies to recognize and apply the best available evidence or not to act in the absence of it, as has been previously reported [45].

It is important to note that these practices were also favored by the Peruvian Ministry of Health (N°270-2020-MINSA), which recommended the use of ivermectin, azithromycin, and hydroxychloroquine to treat cases of mild COVID-19 [46]. More than 50% of our population used these recommendations as a guide for their decision-making, which has presented unclear methodologies and without a basis in quality scientific evidence [47]. However, even though the guidelines expired at the end of 2020 [48], there was still a considerable frequency of prescription drugs for mild COVID-19 in 2021, a phenomenon observed in other pandemics [49].

Among the most prescribed drugs, we found a high frequency of prescription of azithromycin, followed by ivermectin, other antibiotics, and dexamethasone. This is in agreement with what was reported in Italy [42], Pakistan [11] and different studies in Latin America [10,50], where a higher prescription of antibiotics such as azithromycin was found for the management of patients with COVID-19. This high prescription of antibiotics is to be expected, since it is a common practice for the management of respiratory infections, even before the pandemic [51,52], mainly offered with the intention of “avoiding complications” [53], and because of the pressure exerted by patients on professionals for their consumption [54]. The case of azithromycin becomes a particular situation since it was postulated that it may have an immunomodulatory effect on the disease, but this was later not corroborated [55] and its use could result in greater bacterial resistance and a reduced supply for other diseases, especially in rural communities where the majority of recently graduated physicians work [56]. Both the prescription of ivermectin and dexamethasone may be due to the perception of their benefit in preventing complications of the disease and consequently hospitalization and death [57]. These drugs were promoted in the media and by physicians with public influence in Peru [37], which led to massive purchases and promotion of their use in primary care centers, where our surveyed population mainly works [58].

A paradoxical situation observed in our research is the low prescription of hydroxychloroquine, considering that in developed countries such as the United States [59], the opposite situation was observed. In this country (United States) hydroxychloroquine was one of the predominantly prescribed drugs due to the results of studies with low methodological quality [60] and the public endorsement of the drug by the President of the United States. In contrast, in our context, the low prescription is possibly due to its lower availability and higher cost [61,62].

### 5.3 Competences in EBM and drug prescription

Approximately one out of every two physicians included rated all their competencies in EBM as inadequate, with “defining and asking a scientific question”, “judging the quality of the information” and “identifying the possible implications” being the competencies with the lowest perceived expertise. Previous studies have corroborated that there is a greater perception of difficulty for physicians in these dimensions [63]. Multiple reasons can be postulated to explain these findings, one of them being that educational interventions in EBM are mainly focused on teaching theoretical classes on critical evaluation of evidence and information search [63,64], leaving aside the other dimensions. Therefore, subjects such as epidemiology and biostatistics are necessary and should be emphasized at the undergraduate level to consolidate competencies in EBM. On the other hand, studies mention that EBM is a practical application of methodological knowledge [65], which is known to be deficient in undergraduate medicine in Peru [66,67], leading to an imbalance in the learning of the dimensions of EBM, mainly in those that imply greater practice, such as those mentioned above.

A study of recently graduated Peruvian physicians during the pandemic concluded that they had a low self-perception of having acquired competencies to perform EBM [63], as did our study. In Peru, research teaching has several shortcomings in terms of the structure of curricular plans, which do not include rigorous EBM or research courses, include teachers with little experience in research, and there is little investment in this area [68,69]; all of which should be addressed and prioritized by educational institutions. However, despite the findings, it is important to take into account that our study has measured competencies under self-perception, which can be found to be directed to report better EBM competencies [70], overestimating our results. Some authors have even shown that self-perception levels are higher when knowledge and experience in clinical practice are lower [71], as in this population group.

Lack of access to resources to find quality evidence (databases, journals) and lack of logistic resources (medical equipment or technologies) were two of the four most reported reasons why physicians did not apply EBM in their clinical practice. This is one of the most reported barriers in different systematic reviews on the subject [72,73]. This logistic barrier likely leads many physicians to still use textbooks and the opinions of their colleagues as the most reliable resources to answer their clinical questions in daily practice instead of e-books based on the best available evidence at the time of consultation. This practice could introduce interpretations biased by logical fallacies when using arguments from authority [74]. In Peru, some bibliographic resources can be accessed through registration with the National Council of Science, Technology, and Innovation (CONCYTEC) and some universities that have subscriptions [69]. However, few physicians can access these resources because they are only available to selected researchers and academic institutions, and may face the ethical dilemma of resorting to illegal means to access information, such as Sci-Hub [75]. Therefore, it is recommended to increase access to these resources, something that has been promoted by the improvement of university quality in recent years in Peru and should continue [69,75].

Considering general EBM competencies as adequate was associated with less prescribing of drugs without evidence against mild COVID-19. The steps during the EBM process are the formulation of a clinical question, information search, critical analysis, application, and evaluation [76]. The completion of these steps with adequate skill and integration of their components, as well as, previous clinical experience and consideration of the patient's perspective [77], determine better clinical judgment by using the best available evidence [78]. This association between EBM competencies and prescribing that we found is important because in the Peruvian health context there is inadequate distribution of human resources in health. Recently graduated physicians, in many cases, are the only source of care available for many rural and marginalized populations [79]. Therefore, poor quality of care, consequent to the lack of application of EBM, could undermine the health and quality of life of these populations and thus perpetuate inequalities [80].

On the other hand, EBM is not perfect, as it can limit clinical judgment, lead to mechanical reasoning, and lead to overconfidence in the reliability of clinical trials and systematic reviews [81]. It can also lead to adverse effects such as relegating less tangible values, such as knowledge creation and patient experience and dignity. In this context, EBM cannot be only about drugs or devices, it must be willing to question and challenge all aspects of medicine and health care, whether commercial, ideological, or political [81].

When we analyzed the association of medication prescribing with each of the specific EBM competencies, we found that not all components are associated with prescribing and even one component could be associated with increased prescribing. In this regard, it should be taken into account that, when analyzing each component separately, respondents' self-ratings may be biased and that in many cases they do not have adequate competence in the other domains. This would highlight the importance of acquiring EBM competencies as a whole since to reach an adequate clinical judgment, it is necessary to integrate all aspects of EBM, recognizing the limitations that may exist in some scenarios. Likewise, it should be recognized that the statistical power for most of the components was less than 80%; therefore, research is needed that considers a better sample size and probability sampling to define more precisely the role of EBM knowledge in the prescription of drugs that have negative evidence for their use.

It is necessary to strengthen the initiatives of public institutions in the country that promote evidence-based decision decision-making is also necessary to strengthen public health institutions so that they begin to integrate EBM competencies. Finally, it is a priority to deploy efforts in the education of medical students so that they acquire the necessary basic competencies of evidence-based medicine in their future work as physicians [63]. Therefore, it is necessary to reformulate the contents of the curricula of the different medical schools to include training aimed at efficiently using and interpreting the various health-related information resources, to achieve a correct application of this knowledge for decision-making in patient care [20]. In addition, it is important to provide access to resources such as specialized databases. Finally, it would be possible to promote the formation of research seedbeds within the universities that provide the subjects and mentoring that help to form all the competencies of EBM.

### 5.4 Limitations and strengths

The present study has certain limitations that must be taken into account. First, there is a lack of temporality due to the cross-sectional design. Secondly, non-probability sampling was used to enroll the participants, which makes it difficult to extrapolate the results. In addition, the fact that the survey was virtual and distributed through social networks contributes to selection bias. This is reflected in that the majority of the sample was predominantly from the first period of SERUMS and from the highlands. Third, the statistical power for associations that were not significant was low, implying that the lack of association may be due to lack of sample size. Fourth, because the survey was based on self-report, participants' ratings were given based on their responses; therefore, participants with lack of instruction in EBM may have had a biased self-rating by overestimating their ability (cognitive bias known as the Dunning-Kruger effect) [82]. However, it is presumed that this bias did not significantly affect our results because less medication prescription was found in those participants who perceived their EBM skills as adequate. Finally, collecting information on prescribing practices during the last two years exposes our results to recall bias.

Despite these limitations, to our knowledge this is the first study to evaluate the association between EBM competencies and the prescription of drugs without quality scientific evidence to treat mild cases of COVID-19. In addition, the population studied spans different departments of Peru and analyzes the evolution of drug prescribing over two SERUMS periods during the COVID-19 pandemic.

## 6 Conclusions

In conclusion, seven out of ten physicians evaluated prescribed some type of medication without scientific evidence to treat patients with mild COVID-19. The prescribing trend was decreasing between 2020 and 2021. The most prescribed medications were ivermectin, azithromycin, other antibiotics, and dexamethasone. Approximately half of the respondents self-rated the assessed domains on EBM as adequate. The main reason for having limited application of EBM in clinical practice was lack of access to resources to find quality evidence. Having adequate self-perceived EBM competencies was associated with a lower frequency of prescribing medications without scientific evidence to manage patients with mild COVID-19. Therefore, and with the aim of reducing inappropriate prescribing of drugs, it is necessary to prospectively assess EBM competencies, with validated instruments, at different stages of physicians. In addition, it is necessary to assess whether inadequate EBM competencies could influence unjustified prescribing in other diseases.

## Author contribution statement

Daniel Fernandez-Guzman: Conceived and designed the experiments; Performed the experiments; Analyzed and interpreted the data; Contributed reagents, materials, analysis tools or data; Wrote the paper. Brenda Caira-Chuquineyra; Wendy Nieto-Gutierrez; Vicente A. Benites-Zapata: Performed the experiments; Analyzed and interpreted the data; Contributed reagents, materials, analysis tools or data; Wrote the paper. Fiorella Baca-Rondan; Maria Cristina Yucra-Sosa: Conceived and designed the experiments; Contributed reagents, materials, analysis tools or data; Wrote the paper. Fabricio Ccami-Bernal; David R. Soriano-Moreno: Contributed reagents, materials, analysis tools or data; Wrote the paper.

## Data availability statement

Data will be made available on request.

## Declaration of interest's statement

The authors declare no conflict of interest.

## Additional information

Supplementary content related to this article has been published online at [URL].

## Funding statement

The research received partial funding from Universidad Científica del Sur in Lima, Perú.
