# Supplementary material for: Association between self-reported evidence-based medicine competencies and prescribing of drugs without scientific evidence against mild COVID-19 among recently graduated physicians in Peru
Source: Heliyon. 2023 Apr 8;9(4):e15366. doi: 10.1016/j.heliyon.2023.e15366 (PMC10082469; doi:10.1016/j.heliyon.2023.e15366)
Supplement: Multimedia component 1 [file mmc1.docx]

## Supplementary material

**Supplementary Material 1. Figures**

**Figure S1. Use and knowledge of information sources. MINSA: Ministry of Health of Peru (Spanish acronym).**

**Figure S2. Reasons for not applying EBM in clinical practice.**

**Supplementary Material 2. Questionnaire.**

****The original questionnaire is in Spanish***

**GENERAL DATA SECTION**

1. How old are you? ______________

2. What is your gender?

1. Woman
2. Man

3. What is your marital status?

1. Bachelor
2. Married or cohabiting
3. Divorced or Separated
4. Widower

4. Where do you live? (DEPARTMENT)______________________________

5. Where did you do your undergraduate studies?

1. National University
2. Private University

6. During your undergraduate studies, did you belong to any scientific society?

1. No
2. Yes

7. Where did you perform your SERUMS?

1. In a rural area
2. In a marginal urban area
3. In a non-marginal urban area

8. In what type of health center have you been performing your SERUMS?

1. Category I-2
2. Category I-3
3. Category I-4
4. Category II or higher

9. Were you diagnosed with COVID-19?

1. No
2. Yes

**SECTION 2: PRESCRIPTION PATTERNS FOR TREATING COVID-19**

10. During what period of time have you prescribed the following substances to "TREAT" mild or moderate COVID-19 infection (check the 1st and last month you prescribed them at least once)?

| 1. Ivermectin | a) Never  b) During SERUMS in 2020  c) During SERUMS in 2021 |
| --- | --- |
| 2. Azithromycin | a) Never  b) During SERUMS in 2020  c) During SERUMS in 2021 |
| 3. Paracetamol | a) Never  b) During SERUMS in 2020  c) During SERUMS in 2021 |
| 4. NSAIDs | a) Never  b) During SERUMS in 2020  c) During SERUMS in 2021 |
| 5. Hydroxychloroquine | a) Never  b) During SERUMS in 2020  c) During SERUMS in 2021 |
| 6. Dexamethasone | a) Never  b) During SERUMS in 2020  c) During SERUMS in 2021 |
| 7. Oral anticoagulants | a) Never  b) During SERUMS in 2020  c) During SERUMS in 2021 |
| 8. Other antibiotics | a) Never  b) During SERUMS in 2020  c) During SERUMS in 2021 |
| 9. Chlorine dioxide | a) Never  b) During SERUMS in 2020  c) During SERUMS in 2021 |

11. What was the main reason you decided to use these substances as a treatment for mild to moderate COVID-19 infection?

1. I did not use medication
2. Because the Ministry of Health recommended it
3. Because there was coercion to prescribe them at your workplace
4. Because patients asked me to prescribe them for them
5. Because other physicians recommended them
6. Because the existing evidence seemed to demonstrate their effectiveness

12. As a treatment for patients with mild or moderate COVID-19, what is your opinion about the use of the following medications?

| 1. Ivermectin | a) It is effective and does good  b) It is effective, but does harm  c) It is not effective, but does no harm  d) It is not effective and does harm  e) I am not informed on this subject |
| --- | --- |
| 2. Azithromycin | a) It is effective and does good  b) It is effective, but does harm  c) It is not effective, but does no harm  d) It is not effective and does harm  e) I am not informed on this subject |
| 3. Paracetamol | a) It is effective and does good  b) It is effective, but does harm  c) It is not effective, but does no harm  d) It is not effective and does harm  e) I am not informed on this subject |
| 4. NSAIDs | a) It is effective and does good  b) It is effective, but does harm  c) It is not effective, but does no harm  d) It is not effective and does harm  e) I am not informed on this subject |
| 5. Hydroxychloroquine | a) It is effective and does good  b) It is effective, but does harm  c) It is not effective, but does no harm  d) It is not effective and does harm  e) I am not informed on this subject |
| 6. Dexamethasone | a) It is effective and does good  b) It is effective, but does harm  c) It is not effective, but does no harm  d) It is not effective and does harm  e) I am not informed on this subject |
| 7. Oral anticoagulants | a) It is effective and does good  b) It is effective, but does harm  c) It is not effective, but does no harm  d) It is not effective and does harm  e) I am not informed on this subject |
| 8. Other antibiotics | a) It is effective and does good  b) It is effective, but does harm  c) It is not effective, but does no harm  d) It is not effective and does harm  e) I am not informed on this subject |
| 9. Chlorine dioxide | a) It is effective and does good  b) It is effective, but does harm  c) It is not effective, but does no harm  d) It is not effective and does harm  e) I am not informed on this subject |

**SECTION 3: CLINICAL PRACTICE CHARACTERISTICS AROUND CLINICAL PRACTICE**

13. Have you received any training on evidence-based medicine during your medical training?

1. No
2. Yes

14. Have you ever used the evidence-based medicine approach to prescribe COVID-19 treatments?

1. No
2. Yes

15. What was the main basis for your clinical practice in caring for patients with COVID-19?

1. Information I obtain from attending courses or conferences
2. Information I get from national policies and protocols
3. Information that I obtain from scientific articles or medical resources
4. Information I get from the media
5. Information I obtain from social networks

16. As a SERUMS physician, how often did you consult the following sources of information to care for patients with COVID-19?

| 1. To medical colleagues within your health care facility. | a) Never  b) Rarely  c) Sometimes  d) Often  e) Always |
| --- | --- |
| 2. To medical colleagues outside of your health center. | a) Never  b) Rarely  c) Sometimes  d) Often  e) Always |
| 3. Research published in scientific journals | a) Never  b) Rarely  c) Sometimes  d) Often  e) Always |
| 4. Clinical Practice Guidelines | a) Never  b) Rarely  c) Sometimes  d) Often  e) Always |
| 5. Other medical resources (uptodate, clinicalkey, medscape) | a) Never  b) Rarely  c) Sometimes  d) Often  e) Always |
| 6. MINSA Recommendations | a) Never  b) Rarely  c) Sometimes  d) Often  e) Always |
| 7. Socials networks (including medical content pages) | a) Never  b) Rarely  c) Sometimes  d) Often  e) Always |

17. What scientific journal, databases or medical resources did you use to make clinical decisions about COVID-19 during your SERUMS year?

|  | I do not know it | I know it, but do not use it | I know it and use it infrequently | I know it and use it frequently |
| --- | --- | --- | --- | --- |
| Pubmed |  |  |  |  |
| SCOPUS |  |  |  |  |
| Web of Science |  |  |  |  |
| SCIELO |  |  |  |  |
| Cochrane |  |  |  |  |
| Google (Including Google scholar) |  |  |  |  |
| International scientific journals |  |  |  |  |
| National scientific journals |  |  |  |  |
| Medscape |  |  |  |  |
| Uptodate |  |  |  |  |
| ClinicalKey |  |  |  |  |
| Clinical Practice Guidelines |  |  |  |  |
| MINSA Recommendations |  |  |  |  |

18. What is your opinion about evidence-based medicine?

|  | Strongly Disagree | Disagree | Neutral | Agree | Strongly Agree |
| --- | --- | --- | --- | --- | --- |
| EBM cannot be applied to all patients. |  |  |  |  |  |
| EBM threatens clinical freedom or clinical judgment |  |  |  |  |  |
| EBM ignores the values and preferences of patients |  |  |  |  |  |
| EBM is a tool to reduce health care costs |  |  |  |  |  |
| EBM is limited to researchers |  |  |  |  |  |
| EBM would improve my clinical practice |  |  |  |  |  |

19. How would you rate your competencies on the following topics in EBM?

|  | Very inadequate | Inadequate | Neutral | Adequate | Very Adequate |
| --- | --- | --- | --- | --- | --- |
| Defining and asking a scientific question |  |  |  |  |  |
| Finding quality scientific research |  |  |  |  |  |
| Understanding scientific research information |  |  |  |  |  |
| Judging the quality of scientific research information |  |  |  |  |  |
| Identifying possible implications of scientific research |  |  |  |  |  |
| Applying evidence to practice |  |  |  |  |  |

20. What obstacles did you face or would you face in changing your clinical practice to an evidence-based practice? (You can check more than one option)

- Lack of education in EBM
- Lack of English language proficiency
- Lack of authority in the place where I work
- Lack of logistical resources (e.g., new medical technologies)
- Lack of access to resources to find quality evidence (databases, journals, etc.)
- Lack of applicability of evidence to individualized patient care
- Lack of patient acceptance of new clinical practices
- Lack of time
